# Serum hepcidin measurement is related to clinical parameters and interferon-gamma in anemia of inflammation

**DOI:** 10.31744/einstein_journal/2026AO1756

**Published:** 2026-06-23

**Authors:** Raysa Oliveira Maciel, Priscila da Silva Mendonça, Anacélia Gomes de Matos Mota, Deysi Viviana Tenazoa Wong, Roberto César Pereira Lima, Ronald Feitosa Pinheiro, Silvia Maria Meira Magalhães

**Affiliations:** 1 Universidade Federal do Ceará Cancer Cytogenomic Laboratory Fortaleza CE Brazil Cancer Cytogenomic Laboratory, Universidade Federal do Ceará, Fortaleza, CE, Brazil.; 2 Universidade Federal do Ceará Post-graduate Program in Medical Science Fortaleza CE Brazil Post-graduate Program in Medical Science, Universidade Federal do Ceará, Fortaleza, CE, Brazil.; 3 Universidade Federal do Ceará Hospital Universitário Walter Cantídio Fortaleza CE Brazil Hospital Universitário Walter Cantídio, Universidade Federal do Ceará, Fortaleza, CE, Brazil.; 4 Universidade Federal do Ceará Department of Clinical Medicine Fortaleza CE Brazil Department of Clinical Medicine, Universidade Federal do Ceará, Fortaleza, CE, Brazil.

**Keywords:** Anemia, Inflammation, Cytokines, Interferongamma, Hepcidins

## Abstract

**Objective:**

This study aimed to assess the relationship between hepcidin, IFN-γ, iron biomarkers, and clinical variables in patients with anemia of inflammation.

**Methods:**

A cross-sectional study was conducted. Ninety patients with inflammatory anemia and 70 healthy controls were evaluated. Laboratory parameters, serum hepcidin, and IFN-γ were determined using ELISA.

**Results:**

The mean age of patients with anemia of inflammation was 74.1±7.8 years, and most were female. Most patients reported ≥3 comorbidities (70%). Patients with ≥3 comorbidities had significantly higher levels of hepcidin (p<0.001). Hemoglobin, transferrin saturation index, and serum iron levels were significantly lower in patients with anemia of inflammation than in the control group (p<0.001). The erythrocyte sedimentation rate and ferritin and hepcidin levels increased in the anemia group (p<0.05). A significant positive correlation was observed between hepcidin and ferritin (r=0.372; p=0.001) and IFN-γ (r=0.228; p=0.004). Conversely, a significant negative correlation was found between hepcidin and hemoglobin (r=-0.355; p<0.001).

**Conclusion:**

Understanding how inflammatory parameters and upregulated hepcidin levels act as biomarkers to assess iron homeostasis in anemia of inflammation may be effective for differential diagnosis and therapeutic approaches, such as iron supplementation.

## In Brief

Maciel et al. conducted a cross-sectional study involving 90 patients with anemia of inflammation and 70 healthy controls. Hepcidin and IFN-γ were increased in the anemia of inflammation group. A significant positive correlation was observed between hepcidin and ferritin and IFN-γ. However, a significant negative correlation was found between hepcidin and hemoglobin.

## Highlights

■ One-third of patients older than 65 years exhibit a hyperinflammatory state typical of inflammatory anemia.
■ The pathophysiology of anemia of inflammation is multifactorial.
■ Higher inflammation is associated with high prevalence of anemia and age-associated disorders.
■ The inflammatory state stimulates hepatocytes to produce hepcidin.
■ Understanding the immunomodulatory parameters in anemia of inflammation may be effective for differential diagnosis and therapeutic approaches.

## Graphical abstract

**Figure:**
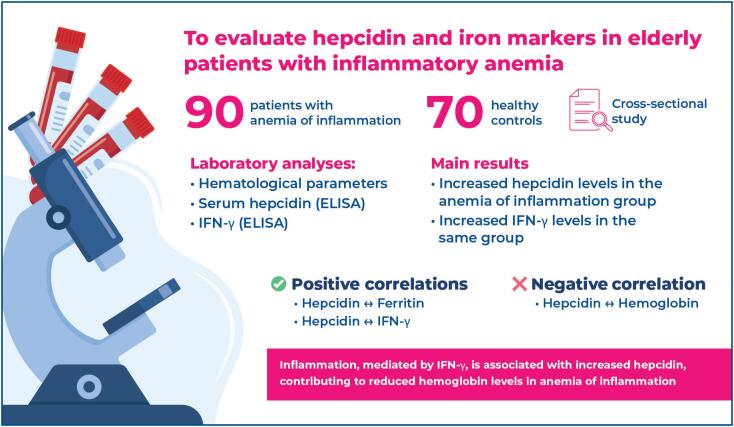

## INTRODUCTION

Anemia is a pathological condition associated with frailty, increased morbidity and mortality, and reduced quality of life, particularly in elderly individuals. A wide range of causes and underlying diseases are known to cause anemia in older adults. At least one-third of patients older than 65 years exhibit a hyperinflammatory state typical of anemia of inflammation, which is considered the second most prevalent cause of anemia, after nutritional deficiency anemia. Diagnosis may be challenging, particularly in cases of coexisting conditions.^(1,2)^

The pathophysiology of anemia of inflammation is multifactorial and includes reduced erythrocyte survival due to direct damage to erythroid progenitors through the formation of oxygen radicals or induction of apoptosis, low levels of erythropoietin, downregulation of EPO receptor expression on erythroid progenitors, and iron metabolism abnormalities. These mechanisms are driven by the immune response and inflammation. Most of these abnormalities in iron metabolism can be explained by the upregulation of hepcidin induced by increased inflammatory cytokines.^(3)^

Due to immunosenescence, stimulated immune cells, such as macrophages and T lymphocytes, abnormally express pro-inflammatory cytokines, such as IL-6, TNF-α, and IFN-γ, resulting in a low-grade chronic inflammatory condition, known as inflammaging. This inflammatory state explains the high prevalence of anemia and its associated disorders.

This inflammatory state stimulates hepatocytes to produce hepcidin, a hormone that regulates iron homeostasis.^(4-6)^ Hepcidin reduces duodenal iron absorption and release from macrophages, leading to iron-restricted erythropoiesis.^(7-12)^

To the best of our knowledge, there are no studies evaluating the relationship between IFN-γ expression and hepcidin in patients with anemia of inflammation.

## OBJECTIVE

This study aimed to evaluate serum hepcidin levels, standard biochemical markers of iron metabolism, and their relationship with clinical and laboratory variables in older patients with anemia of inflammation at a tertiary university center.

## METHODS

### Study population

The study included 160 individuals including 90 patients with anemia of inflammation (aged ≥60 years old) and 70 older healthy controls from a single tertiary university institution. Data were collected between January 2020 and December 2021. All patients newly diagnosed with anemia of inflammation during the study collection period were included. All healthy control participants were over 60 years old and exhibited normal hematological parameters.

Anemia was defined according to the WHO criteria as follows: hemoglobin <12g/dL for women and less than 13g/dL for men.^(13)^ In addition, low transferrin saturation, normal or higher serum ferritin levels, and low total iron-binding capacity have been used as diagnostic criteria for inflammatory anemia.

Patients were classified into three groups according to hemoglobin level: mild anemia (Hb 10.00-12.0g/dL in women and 10.0-13.0g/dL in men), moderate anemia (Hb 8.0- 9.9g/dL), and severe anemia (Hb 6.5-7.9g/dL).

This study was approved by the local ethics committee of the *Universidade Federal do Ceará* (CAAE: 25981919.2.0000.5054; # 3.817.121). Written informed consent was obtained from all the participants.

### Data collection

Demographic, clinical, and comorbidity data were collected from medical records. Polypharmacy was defined as the regular use of at least five medications. Habits such as alcohol consumption and smoking were self-reported by the patients.

### Inflammatory markers

Serum samples were obtained from patients with anemia of inflammation after a 12-hour fast and stored at −80 ºC. Serum levels of hepcidin and IFN-γ, classically considered as a pro-inflammatory cytokine, were both determined by sandwich enzyme-linked immunosorbent assay kit (ELISA) according to the manufacturer's protocol (Invitrogen, Carlsbad, CA, USA).

The concentrations of hepcidin and IFN-γ were determined by spectrophotometric measurement of absorbance at 450nm. Curve-fitting software was used to construct a standard calibration curve ranging from 0 to 250ng/mL, which was subsequently used to quantify cytokine levels in the test samples. All measurements were conducted in duplicate and the results were expressed in nanogram per milliliter (ng/mL).

### Anemia of inflammation diagnosis

Given that anemia of inflammation is often a diagnosis of exclusion, all patients were subjected to a local protocol to rule out nutritional deficiency anemia, hemolysis, hormonal dysfunction, viral infections, and metabolic causes. Bone marrow was analyzed when relevant. Therefore, the diagnosis of anemia of inflammation was confirmed based on clinical features and a comprehensive laboratory evaluation.

The diagnosis of inflammatory anemia was established based on the presence of normocytic and normochromic anemia, accompanied by characteristic laboratory findings, including decreased serum iron levels, reduced transferrin saturation, elevated serum ferritin concentrations, suppressed erythropoietin (EPO) levels, and increased inflammatory markers such as C-reactive protein (CRP) and erythrocyte sedimentation rate (ESR).^(14)^

### Statistical analysis

Normality was verified using the Shapiro-Wilk test. Student's *t*-test, Levene's test, Pearson's χ^2^ test, Fisher's exact test, and Pearson's Correlation were performed using SPSS® (SPSS Inc., Chicago, IL, USA) software version 20.0. A *p<0.05* was considered significant.

## RESULTS

Seventy percent of patients in the inflammation group were female (78.8%). The mean age of patients was 74.1±7.8 years. Polypharmacy, defined as the regular use of five or more medications, was reported in 64 patients (71%). Only 10 patients (11%) had previously undergone transfusion.

Most patients reported three or more comorbidities (70%), including systemic arterial hypertension (90%), diabetes (58%), and dyslipidemia (40%) (Table 1). Patients with ≥3 comorbidities had significantly higher levels of hepcidin than those with <3 comorbidities (48.62ng/mL *versus* 32.65ng/mL, p<0.001).

**Table 1:** Sociodemographic data of elderly patients with anemia of inflammation. (n=90)

Characteristic		n (%)
Gender		
	Male	20 (22)
	Female	70 (78)
Age (years)		
	Mean	74.12 (100)
Age groups		
	60-79	65 (72)
	≥ 80	25 (28)
Transfusion		
	Yes	10 (11)
	No	80 (89)
Alcohol consumption		
	Yes	15 (17)
	No	75 (83)
Smoking		
	Yes	17 (19)
	No	73 (71)
Comorbidities number		
	<3	27 (30)
	≥3	63 (70)
Comorbidities		
	DM	52 (58)
	HAS	81 (90)
	CKD	17 (19)
	Cardiopathy	14 (16)
	Dyslipidemia	36 (40)
Gastrointestinal diseases		9 (10)
	Arthrosis	9 (10)
	Osteopenia	3 (3)
	Rheumatoid arthritis	8 (9)
	Hypothyroidism	12 (13)
	Obesity	13 (14)
	Neoplasm	3 (3)
	COPD	7 (8)
Polypharmacy		
	Yes	64 (71)
	No	26 (29)

HAS: systemic arterial hypertension; CKD: chronic kidney disease; DM: diabetes mellitus; COPD: chronic obstructive pulmonary disease.

Alcohol consumption and smoking habits were reported in 15 (17%) and 17 (19%) patients, respectively (Table 1). These habits did not influence the severity of anemia. No statistically significant differences were observed in Hb (10.75g/dL *versus* 10.30g/dL, p=0.31), iron (71.µg/dL *versus* 62.0µg/dL, p=0.709), TSI (35.9% *versus* 21.8%, p=0.105), ferritin (258.5ng/mL *versus* 194.6ng/mL, p=0.116), EPO (9.75mIU/mL *versus* 11.0mIU/mL, p=0.53) or hepcidin (48.5ng/dL *versus* 42.4ng/dL, p=0.38), when compared to non-smokers and non-alcohol consumers.

Hemoglobin, TSI, and serum iron levels were significantly lower in patients with anemia of inflammation than in the control group (p<0.001). In contrast, ESR, ferritin, and hepcidin levels were increased in the anemia group (p<0.05). No significant differences were observed in other laboratory data (Table 2).

**Table 2:** Laboratory data of controls and patients with anemia of inflammation

Parameters	Group	Mean	Median	SD	p value
Hemoglobin (g/dL)	Patient	10.31	10.70	1.79	< 0.001*
	Control	15.04	15.00	1.56	
MVC (fL)	Patient	87.45	88.15	9.31	0.387
	Control	89.41	90.00	6.28	
MCHC (g/dL)	Patient	32.11	32.00	2.32	0.300
	Control	32.00	32.00	2.16	
ESR (mm)	Patient	69.00	60.00	36.85	< 0.001*
	Control	19.14	20.00	5.74	
TSI (%)	Patient	23.90	22.00	15.45	< 0.001*
	Control	33.47	33.60	6.21	
Serum iron (ug/dL)	Patient	64.13	65.00	24.90	< 0.001*
	Control	99.83	99.75	3.01	
Ferritin (ng/mL)	Patient	219.21	150.00	179,83	< 0.001*
	Control	75.47	79.00	18.05	
Hepcidin (ng/mL)	Patient	43.83	46.49	20.33	< 0.001*
	Control	23.77	23.09	7.05	
IFN-γ (ng/mL)	Patient	72.32	60.01	18.05	< 0.001*
	Control	34.90	33.69	6.41	

Student's *t*-test was utilized to assess relationships.

* p-values less than 0.05.

TSI: transferrin saturation index; ESR: erythrocyte sedimentation rate; MCV: mean corpuscular volume; MCHC: mean corpuscular hemoglobin concentration.a

A difference between serum iron was observed between the three groups with significantly higher levels in mild than moderate anemia (68.97µg/dL *versus* 51.83µg/dL, p=0.017). Erythrocyte sedimentation rate was significantly higher in moderate than in those with mild anemia (57 *versus* 92 mm, p=0.003). No significant differences in other anemia laboratory parameters (hemoglobin level, transferrin saturation index (TSI), mean corpuscular volume (MCV), and mean corpuscular hemoglobin concentration (MCHC]) were found between the groups (p>0.05).

A significant positive correlation was observed between hepcidin and ferritin (r=0.372; p=0.001) and IFN-γ (r=0.228; p=0.001). However, a significant negative correlation was found between hepcidin and hemoglobin (r=-0.355; p<0.001). The correlations between hepcidin levels and other clinical parameters are presented in table 3.

**Table 3:** Correlation between serum hepcidin and laboratory parameters including interferon-gamma in patients with anemia of inflammation

Parameters	Correlation coefficient	p value
Hemoglobin (g/dL)	-0.355	< 0.001*
Ferritin (ng/mL)	0.372	0.001*
IFN-γ (ng/mL)	0.228	0.004*
Age (years)	-0.119	0.267
MCV (fL)	0.055	0.606
TSAT (%)	0.173	0.151
MCHC (g/dL)	0.040	0.709
ESR (mm)	0.086	0.507
EPO (mU/ml)	-0.096	0.672
Creatinine (mg/dL)	0.095	0.375

Pearson's correlation coefficient was used to assess relationships.

* p-values less than 0.05.

MCV: mean corpuscular volume; TSAT: transferrin saturation; MCHC: mean corpuscular hemoglobin concentration; EPO: erythropoietin; IFN-γ: interferon-gamma; ESR: erythrocyte sedimentation rate.

## DISCUSSION

The incidence of anemia increases with age and is a public health problem. Previous studies have reported a higher prevalence in men than in women. However, in our study, most of the patients were female, a trend which has been observed in previous studies. This may be because Brazilian women seek health services more often and earlier than men.^(15,16)^ Additionally, the significantly higher mortality rate in men may play a role in the feminization of old age. Additionally, habits such as smoking and alcohol consumption have been associated with an increased susceptibility to anemia.^(17,18)^ In this study, these habits did not influence the severity of anemia.

Hemoglobin, TSI, and serum iron levels were significantly decreased, whereas the ESR and ferritin levels were elevated in patients with anemia of inflammation. Notably, these alterations are consistent with the pathophysiological profile of anemia of inflammation and likely reflect the diagnostic criteria used, suggesting that the observed differences may constitute a circular confirmation of the case definition rather than a novel or independent finding.

Serum iron and TSI levels were lower in patients with higher serum ferritin levels. An important feature in the pathophysiology of anemia and inflammation is the disturbance of iron homeostasis, with increased iron sequestration and retention within cells of the reticuloendothelial system, restricting iron availability. Hepcidin, which interacts with the transmembrane protein ferroportin, is a crucial regulator of iron homeostasis, and its expression is upregulated by inflammatory cytokines.^(19-20)^ Higher levels of hepcidin are present in individuals with chronic diseases characterized by overt or persistent inflammation.^(21,22)^

In this study, serum hepcidin was significantly higher in the anemia of inflammation group and higher in subjects with ≥3 comorbidities. Several studies have reported on cytokine expression in patients with inflammation. However, few studies have evaluated the relationship between IFN-γ expression and hepcidin levels in patients with anemia of inflammation. The positive correlation between hepcidin and IFN-γ supports the hypothesis that IFN-γ contributes to the inflammatory processes seen in anemia of inflammation by increasing the synthesis and release of hepcidin, leading to iron-restricted erythropoiesis and contributing to the promotion and progression of anemia in these patients.^(23)^

However, this study has some limitations. The variety of comorbidities of anemia in the inflammation group and the concomitant presence of more than one disease did not make it possible to evaluate the impact of each disease alone. Moreover, the integration of multivariate analyses and multicenter studies may offer additional valuable insights into research involving inflammatory anemia.

## CONCLUSION

Higher levels of hepcidin were associated with the presence of multiple comorbidities and positively correlated with IFN-γ expression. Therefore, the increased levels of IFN-γ and hepcidin in patients with anemia of inflammation presupposes their role in the pathogenesis of anemia in these individuals.

Understanding how inflammation and the subsequent upregulation of hepcidin function as biomarkers may be effective for improving the accuracy of differential diagnosis, monitoring of iron supplementation, and potentially highlighting new targets for therapeutic approaches to anemia of inflammation.

## Data Availability

The datasets generated during the current study are available from the main author upon reasonable request.
